# MicroRNA in neuroexosome as a potential biomarker for HIV-associated neurocognitive disorders

**DOI:** 10.1007/s13365-024-01241-8

**Published:** 2025-01-16

**Authors:** Kotaro Arizono, Ayako Sedohara, Khulan Tuvshinjargal, Takahiro Tanaka, Michiko Koga, Fumio Nakahara, Amato Ootani, Yoshiaki Kanno, Kazuhiko Ikeuchi, Makoto Saito, Eisuke Adachi, Takeya Tsutsumi, Hiroshi Yotsuyanagi

**Affiliations:** 1 Department of Computational Biology and Medical Sciences, Graduate School of Frontier Sciences, The University of Tokyo, 7-3-1, Hongo, Bunkyo-Ku, Tokyo 113-8654 Japan; 2 Division of Infectious Diseases, Advanced Clinical Research Center, Institute of Medical Science, The University of Tokyo, 4-6-1, Shirokanedai, Minato-Ku, Tokyo 108-8639 Japan; 3 Department of Infectious Disease and Applied Immunology, IMSUT Hospital of The Institute of Medical Science, The University of Tokyo, 4-6-1, Shirokanedai, Minato-Ku, Tokyo 108-8639 Japan; 4 Department of Infectious Diseases, Faculty of Medicine, The University of Tokyo, 7-3-1, Hongo, Bunkyo-Ku, Tokyo 113-8654 Japan; 5 Division of Regenerative Medicine, Center for Molecular Medicine, Jichi Medical University, 3311-1, Yakushiji, Shimotsuke-Shi, Tochigi 329-0498 Japan

**Keywords:** HIV-associated neurocognitive disorder (HAND), Neuronal-derived exosomes, Neuroexosomes, miRNA, Biomarkers

## Abstract

HIV-associated neurocognitive disorder (HAND) is a complication of chronic inflammation caused by HIV infection that impairs cognitive and motor functions. HAND can occur at any age, regardless of the duration of infection, even in people living with HIV (PLWH) whose blood viral load is controlled by antiretroviral therapy. The diagnosis of HAND requires a battery of neuropsychological tests, which is time-consuming and burdensome, limiting its effectiveness for screening PLWH. Here, we aimed to identify biomarkers for quantitatively diagnosing and screening for HAND using minimally invasive blood tests. Neuronal-derived exosomes (neuroexosomes) were isolated from the peripheral blood of PLWH, and the transcriptomes of their microRNAs (miRNAs) were analyzed. We identified five upregulated miRNAs (hsa-miR-16-5p, hsa-miR-26a-3p, hsa-92a-3p, hsa-miR-103a-3p, and hsa-miR-185-5p), and two downregulated miRNA (hsa-miR-3613-3p and hsa-miR-4668-5p) in PLWH diagnosed with HAND (HAND PLWH). Functional analysis of five miRNAs whose expression levels increased in HAND PLWH using the database showed that these miRNAs are involved in motor proteins and endocytosis, which are associated with nerve function. The expression levels of hsa-miR-16-5p, hsa-miR-103a-3p, and hsa-miR-185-5p were significantly higher than those in the non-HIV controls and non-HAND PLWH, suggesting that these miRNAs are potential biomarkers for HAND. Since there were no changes in known dementia miRNA biomarkers in HAND PLWH, the miRNAs identified in this study will allow for early differentiation of HAND.

**Supplementary Information:**

The online version contains supplementary material available at 10.1007/s13365-024-01241-8.

## Introduction

HIV is an RNA virus belonging to the Retroviridae family that infects CD4 + T cells and controls cellular and humoral immunity. The inhibition of HIV replication by antiretroviral therapy (ART) has improved the prognosis of individuals infected with HIV, allowing their life expectancy to be similar to that of uninfected individuals (Frescura et al. 2022). However, HIV is incorporated into the human genome, becoming a provirus; thus, even if its multiplication can be suppressed by ART, HIV-infected cells themselves cannot be eliminated. Persistent HIV infection causes chronic inflammation, which in turn leads to various complications, such as cardiovascular disease and abnormal lipid metabolism (Deeks 2011). In particular, HIV-associated neurocognitive disorder (HAND) impairs peoples' living with HIV (PLWH) cognitive, executive, and motor functions and can severely reduce quality of life (Alford et al. 2022; Antinori et al. 2007). The diagnostic criteria for HAND are based on the Frascati criteria (Antinori et al. 2007), which classifies cases according to severity into asymptomatic neurocognitive impairment (ANI), mild neurocognitive disorder (MND), and severe HIV-associated dementia (HAD). Treatment for HAND generally involves antiviral medications that are more easily transferred to the brain, and early intervention can improve neurocognitive function (Underwood and Winston 2016). HAND occurs regardless of age, duration of infection, or length of ART treatment, and an increasing number of PLWH present with cognitive impairment despite their blood viral loads being controlled (Heaton et al. 2011). As cognitive decline makes it difficult to maintain medication adherence and can cause viremia, it is important to provide treatment before the onset of neurocognitive impairment. However, HAND is difficult to diagnose radiologically using computed tomography and also functionally using common dementia screening tests (Ances and Hammoud 2014; Valcour 2011). Diagnosis of HAND requires formal cognitive function assessment, using a battery of neuropsychological tests (NP battery, Kinai et al. 2017). Despite its widespread use, NP battery testing for HAND is time-consuming and burdensome for PLWH, meaning that it is not practical to screen large numbers of PLWH at the clinic. Therefore, such testing is only performed if the physician deems it necessary during consultation or at the PLWH's request, making early intervention in HAND difficult. Identifying specific biomarkers would facilitate the development of minimally invasive blood tests for screening and allow intervention before the development of neurological symptoms.

In recent years, the search for biomarkers that target factors contained in exosomes has progressed (Lai et al. 2022). Exosomes are vesicles 30–150 nm in diameter that contain proteins and nucleic acids, such as microRNAs (miRNAs) and messenger RNAs (mRNAs) (Pegtel et al. 2010), and are responsible for signal transduction between cells (Lee et al. 2012). Exosomes are secreted by almost all cells and are involved in many biological and physiological processes, such as immune response regulation, immune tolerance induction (Admyre et al. 2007), tissue repair (Kadota et al. 2020), and neural transmission (Asai et al. 2015). miRNAs are noncoding RNAs consisting of 18–25 nucleotides that bind to target mRNAs and inhibit translation by promoting degradation (Dong et al. 2013). Defects in miRNA expression are associated with various diseases, including cancer and coronary artery disease (Angelucci et al. 2019; Chandan et al. 2019; Divakaran and Mann 2008; Hansen and Obrietan 2013; Reddy 2015).

Exosomes secreted from the central nervous system (CNS) act as signaling pathways between nerves, some of which cross the cerebral blood barrier and circulate in the peripheral blood (Matsumoto et al. 2017). Significant reductions in miRNA-132 and miRNA-212 levels have been reported in neuroexosomes from patients with Alzheimer’s disease (AD) (Cha et al. 2019). Furthermore, a large-scale screening of 1601 patients with AD, cerebrovascular dementia, and Lewy body dementia identified 78 different miRNAs reflecting their respective dementias (Shigemizu et al. 2019). Thus, several neurological disease-specific miRNAs that are expected to serve as biomarkers have been identified in blood exosomes. Dementia includes Alzheimer's disease (AD), vascular dementia (VD), dementia with Lewy bodies (DLB), frontotemporal dementia (FTLD), Parkinson's disease dementia (PDD), and Parkinson's disease (PD). Transcriptome analysis of miRNAs, including microarray and next-generation sequencing, in neuronal-derived exosomes (neuroexosomes) revealed several miRNAs whose expression increased or decreased specifically in these dementias (Dong et al. 2020). Among these miRNAs, only a few genes are commonly altered in individuals with dementia. hsa-miR-22, −23a-3p, 125b-5p, and 135a are commonly increased in AD and PD/PDD, and hsa-miR-135a is commonly increased in AD, PD, and VD. To understand the pathogenesis of HAND and compare it with that of dementia, it is necessary to identify the miRNAs that are increased or decreased in the neurogenic exosomes of patients with HAND and explore the functions of these miRNAs. In this study, we aimed to identify miRNAs from neuroexosomes that could serve as HAND biomarkers.

## Materials and methods

### Subjects enrolled in this study

Between January 1st, 2017, and July 31st, 2020, seven PLWH diagnosed with HAND (HAND PLWH) (Table 1) and six PLWH non-diagnosed with HAND (non-HAND PLWH) (Table 2) who consented to have their blood drawn for the study after providing informed consent were enrolled in this study. There were no exclusion criteria because only seven of the approximately 500 PLWH admitted to the Institute of Medical Sciences at the University of Tokyo Hospital were diagnosed with HAND. Six non-HAND PLWH enrolled in this study maintained a blood HIV viral load of 20 copies or less for at least three years and had no comorbidities or psychiatric disorders. Five non-HIV controls (healthy volunteers recruited at our facility) were also enrolled in this study (Supplementary Table 1). Non-HIV controls were those who had no fever or other symptoms for 3 days before or after the blood draw and no symptoms of mental illness. As the percentage of HIV-infected people is low in Japan, HIV testing was not performed for the non-HIV controls who cooperated in this study. HAND PLWH were those whose physicians determined that NP battery testing was necessary and who were consequently diagnosed with HAD (*n* = 3), MND (*n* = 2), or ANI (*n* = 1) using the NP battery test. Ethics approval was obtained from the Research Ethics Committee of the University of Tokyo (2023–19–0720). This study complied with the Declaration of Helsinki.

**Table 1:** Clinical information of people living with HIV diagnosed with HAND enrolled in this study

No	Age	Sex	Result of battery test	Date of battery test	Date of blood sampling	HIV-RNA at the time of blood sampling (copies/mL)	CD4 + T-cell at the time of blood sampling (cells/mL)	Nadir CD4 + T-cell (cells/mL)	ART regimen ^b^	Date of HIV diagnosis
P1	29	M	MND	2018.10.24	2017.10.5	57	348	179	TAF,FTC,EVG,cobi	2017.5.17
P2^a^	24	M	HAD	2019.9.25	2018.10.9	47,000	279	192	Untreated	2018.11.26
P3	34	M	HAD	2019.5.8	2019.1.8	58,000	306	255	Untreated	2019.2.15
P4	24	M	MND	2020.5.27	2020.7.22	9,200	297	241	Untreated	2020.5.15
P5	70	M	HAD	2019.4.25	2018.4.20	450,000	58	52	Untreated	2018.4.13
P8	31	M	ANI	2019.9.12	2017.9.5	62	239	128	ABC,3TC,DTG	2012.2.XX^c^
P9	36	M	ANI	2019.8.5	2018.5.28	26	785	221	TAF,FTC,DRV,RTV	2013.1.16

^a^Hearing loss in one ear

^b^*3TC* lamivudine, *ABC* abacavir, *DRV* darunavir, DTG: dolutegravir, FTC: emtricitabine, TAF: tenofovir alafenamide, cobi: cobicistat, EVG; Elvitegravir, RTV: Ritonavir, Untreated: ART treatment had not been started at the time blood samples were collected

^c^Only the year and month in which HIV was diagnosed were recorded

**Table 2:** Clinical information of people living with HIV enrolled in this study

No	Age	Sex	Result of battery test	Date of battery test	Date of blood sampling	HIV-RNA at the time of blood sampling (copies/mL)	CD4 + T-cell at the time of blood sampling (cells/mL)	Nadir CD4 + T-cell (cells/mL)	ART regimen ^b^	Date of HIV diagnosis
P6	46	M	−	−	2017.8.31	140	694	215	TAF,FTC,DTG	2014.5.13
P7	31	M	−	−	2017.9.6	< 20	412	190	TAF,FTC,DRV,RTV	2008.9.XX^c^
P10	37	M	−	−	2017.8.31	< 20	959	68	ABC,3TC,DTG	2007.12.20
P11	59	M	−	−	2017.9.1	< 20	1507	149	TAF,FTC,DTG	2004.4.7
P12	49	M	−	−	2017.9.5	< 20	441	212	TAF,FTC,DTG	1998.6.9
P13	56	M	−	−	2017.9.6	< 20	218	270	ABC,3TC,DTG	2007.3.7

^b^3TC: lamivudine, ABC: abacavir, DRV: darunavir, DTG: dolutegravir, FTC: emtricitabine, TAF: tenofovir alafenamide, RTV: Ritonavir

^c^Only the year and month in which HIV was diagnosed were recorded

### Total exosome extraction from plasma

To remove coagulation factors from plasma, 20 μL of 500 U/mL thrombin (Fujifilm Wako) was added to the plasma. After 10 min, the plasma samples were centrifuged at 9000 × *g* for 10 min at 4 °C. Phosphatase inhibitor cocktail (Thermo) was added to the supernatant, followed by centrifugation at 6000 × *g* for 20 min at 4 °C. After centrifugation, the plasma was collected, and Halt protease and phosphatase inhibitor cocktail (100 ×) (Thermo Fisher Scientific) was added to the plasma and mixed 10 times by inversion, followed by centrifugation at 6000 × *g* for 20 min at 4 °C. Total exosomes were extracted from the supernatant using ExoQuick (System Biosciences) according to the manufacturer’s protocol. Total exosome pellets were dissolved in Dulbecco’s phosphate-buffered saline (Fujifilm Wako Chemicals, Japan).

### NanoSight analysis and electron microscopy

The size and number of exosomes were determined using a NanoSight LM10 instrument (Malvern Instruments, Malvern, UK). Brownian motion images were captured five times for 60 s, with the camera level set to 13. The particle diameters and concentrations were calculated from the videos. For electron microscopy, all the exosomes were dropped onto a 400-mesh grid with a carbon support membrane to disperse the samples. Total exosomes were stained with 2% uranium acetate and observed using a transmission electron microscope (JEOL JEM 1400 Flash).

### Neuroexosome isolation

To isolate neuroexosomes, immunoprecipitation was performed according to previously described methods (Goetzl et al. 2015) using Dynabeads™ Protein G and DynaMag™−2 Magnet (Thermo). The antibodies used for immunoprecipitation are listed in Table 3. To extract proteins from the exosome samples, the exosomes were lysed using RIPA lysis and extraction buffer (Thermo Fisher Scientific) and sonicated using BIORUPTER II (BM) in HIGH mode for 30 s. The protein concentration was measured using the Qubit™ Protein Assay Kit on a Qubit® 3.0 Fluorometer (Thermo Fisher Scientific). The antibodies used for western blotting are listed in Table 3. The effects of each antibody on exosomes or peripheral blood mononuclear cells were examined at appropriate dilutions (Supplementary Fig. 1). SDS‒PAGE and western blotting were performed using an XV PANTERA MP Gel (DRC) and an iBlot 2 Dry Blotting System, respectively, according to the manufacturer’s instructions. To detect CD9 and CD81, which belong to the tetraspanin family, SDS‒PAGE was performed in the absence of 5% 2-mercaptoethanol. The SuperSignal™ West Pico PLUS chemiluminescent substrate was used to detect CD9, CD81, and calnexin, and the SuperSignal™ West Femto maximum sensitivity substrate (Thermo) was used to detect enolase-2. Signals were detected using an iBright FL1500 system (Thermo Fisher Scientific). Protein signals were quantified using iBright Analysis Software ver. 5.2.1.

**Table 3:** List of antibodies used in this study

name	species	clone	applications		dilution	Company (catalog number)
CD9	Mouse	1 K	Western blot		1:2000	FUJIFILM Wako
						(014–27763)
CD81	Mouse	17B1	Immunoprecipitation		−	FUJIFILM Wako
			Western blot		1:2000	(011–27773)
CD63	Mouse	3–13	Immunoprecipitation		−	FUJIFILM Wako
						(012–27063)
L1CAM(CD171)	Mouse	5G3	Immunoprecipitation		−	Thermofisher
						(13–1719-82)
Calnexin	Rabbit	C5C9	Western blot		1:2000	Cell Signaling Technology (2679)
Enolase-2	Rabbit	E2H9X	Western blot		1:1000	Cell Signaling Technology (24330)
APC Mouse IgG2b,	Mouse	MG2b-57	Immunoprecipitation		−	BioLegend (401209)
κ Isotype Ctrl Antibody						
Goat anti-Mouse IgG (H + L)	Goat	− ^a^	Western blot		1:2000	Thermofisher (62–6520)
Secondary Antibody, HRP						
Goat anti-Rabbit IgG (H + L)	Goat	− ^a^	Western blot		Calnexin (1:2000) Enolase-2 (1:1000)	Thermofisher (65–6120)
Secondary Antibody, HRP						

^a^polyclonal antibody

### miRNA extraction and RT‒qPCR

Exosomal miRNAs were extracted using the miRNeasy Mini Kit (QIAGEN) according to the manufacturer’s protocol. The concentration of miRNA was measured using the Qubit™ microRNA Assay Kit (Thermo) on a Qubit® 3.0 Fluorometer (Thermo). A Mir-X miRNA First-Strand Synthesis Kit (Clontech) was used for cDNA synthesis. A Mir-X miRNA RT‒qPCR TB Green Kit (Clontech) was used for qPCR. The primer sequences are listed in Table 4. The mRQ 3' supplied with the Mir-X miRNA RT‒qPCR TB Green Kit was used as a reverse primer. U6 was used as an endogenous control for normalization. The target miRNA was amplified using CFX Connect (Bio-Rad, Hercules, CA, USA) at 95 °C for 10 s (95 °C for 5 s, 60 °C for 20 s) × 50 cycles. The qPCR results were analyzed using the delta-delta Ct (ddCt) method (Livak and Schmittgen 2001).

**Table 4:** Primer sequences

miRNA primer	sequence (5'−3')
hsa-miR-122-3p	AACGCCATTATCACACTAAATA
hsa-miR-124–1-3p	TAAGGCACGCGGTGAATGCCAA
hsa-miR-215-3p	TCTGTCATTTCTTTAGGCCAATA
hsa-miR-16-5p	TAGCAGCACGTAAATATTGGCG
hsa-miR-26a-5p	TTCAAGTAATCCAGGATAGGCT
hsa-miR-92a-3p	TATTGCACTTGTCCCGGCCTGT
hsa-miR-103a-3p	AGCAGCATTGTACAGGGCTATGA
hsa-miR-185-5p	TGGAGAGAAAGGCAGTTCCTGA
hsa-miR-3613-3p	ACAAAAAAAAAAGCCCAACCCTTC
hsa-miR-4668-5p	AGGGAAAAAAAAAAGGATTTGTC
hsa-miR-22	AGTTCTTCAGTGGCAAGCTTTA
hsa-miR23a-3p	ATCACATTGCCAGGGATTTCC
hsa-miR-125b-5p	TCCCTGAGACCCTAACTTGTGA
hsa-miR-135a	CATATGGCTTTTTATTCCTATGTGA

### miRNA microarray

The miRNA concentration and quality were evaluated using a Bioanalyzer 2100 system (Agilent Technologies, Germany). A GeneChip miRNA 4.0 Array (Applied Biosistems^TM^) was used for miRNA transcriptome analysis. miRNA was labeled using Affymetrix® FlashTag™ Biotin HSR RNA Labeling Kits (Affymetrix Inc.) and hybridized for 18 h at 48 °C. The signals were scanned with a GeneChip® Array scanner 3000 7 G (Applied Biosistems^TM^). The miRNA microarray data were analyzed using the Transcriptome Analysis Console (Thermo).

### Prediction of miRNA function

The ceRNA network was constructed using the following miRNA target prediction databases: TargetScan (https://www.targetscan.org/vert_80/, v8.0) (McGeary et al. 2019), miWalk (http://mirwalk.umm.uni-heidelberg.de/, v3.0) (Sticht et al. 2018), miRDB (https://mirdb.org/) (Chen and Wang 2020), ENCORI (https://rnasysu.com/encori/) (Li et al. 2014), and miRTarBase (https://mirtarbase.cuhk.edu.cn/) (Hsu et al. 2011). We then downloaded the experimentally validated lncRNA–mRNA interaction data from the RISE database (http://rise.life.tsinghua.edu.cn/) (Gong et al. 2018). The information on lncRNAs that interact with miRNAs was obtained from the ENCORI database. The PPI network of miRNA‒target mRNAs was analyzed using STRING (https://string-db.org/) (Szklarczyk et al. 2015). Hub genes in the completed PPI network were screened using cytoHubba Ver. 0.1 (Chin et al. 2014). A ceRNA network was constructed using Cytoscape v3.9 software. DAVID (https://david.ncifcrf.gov/) was used for the functional analysis of miRNAs (Sherman et al. 2022), GO analysis of miRNA target mRNAs (Young et al. 2010), and KEGG pathway enrichment analysis (da Huang et al. 2009). The hub genes screened by cytoHubba were also subjected to KEGG pathway enrichment analyses.

### Statistical analysis

Statistical analysis of enolase-2/CD9 and miRNA RT‒qPCR was performed using R (v.4.3.1) (da Huang et al. 2009) and RStudio (version 5.0, Integrated Development for R. RStudio, Inc., Boston, MA, http://www.rstudio.com/). The Shapiro‒Wilk test was used to determine if the data were normally distributed. The Bartlett test was used to verify whether the variances of the experimental groups in the population were equal. Significant differences in the data between experimental groups were verified using the Kruskal‒Wallis test. The Steel–Dwass test was performed to determine whether there were significant differences in the enolase-2/CD9 ratio among the experimental groups. The Steel test was performed to determine if there were significant differences in the qPCR data between the non-HIV control group and the non-HAND PLWH group and between the non-HIV control group and the PLWH diagnosed with HAD.

## Results

### Extracellular vesicles isolated from plasma met the criteria for exosomes

**Fig. 1:**
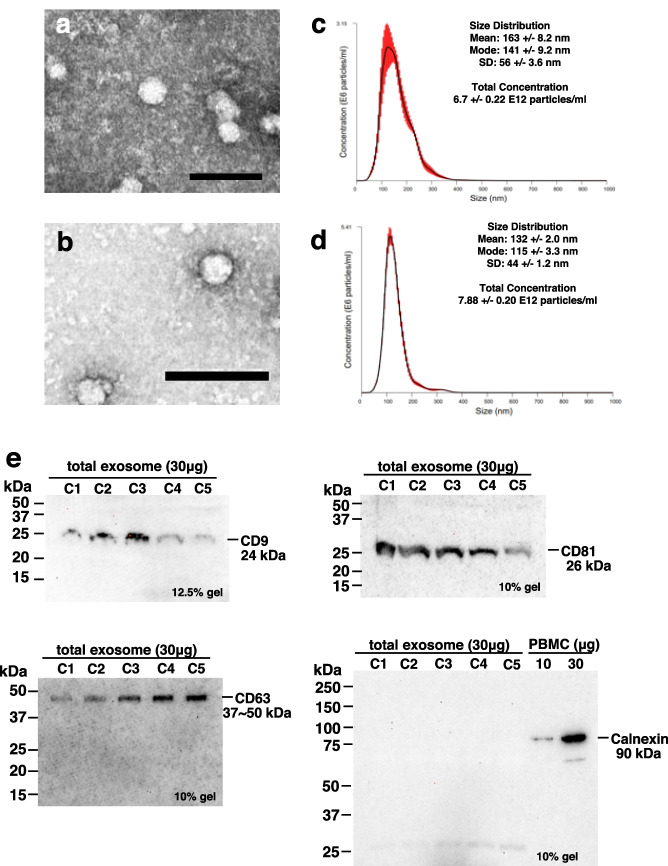
Characterization of total exosomes extracted from plasma. **a** Transmission electron micrographs of total exosomes derived from non-HIV controls. Scale bar, 100 nm. **b** Transmission electron micrographs of total exosomes derived from PLWH diagnosed with HAD. Scale bar, 100 nm. **c** NanoSight analysis of total exosomes derived from non-HIV controls. The black line indicates the means. Red indicates distribution. **d** NanoSight analysis of total exosomes derived from PLWH diagnosed with HAD. The black line indicates the means. Red indicates distribution. **e** Detection of the exosome markers CD9, CD81, and CD63 in total exosomes derived from non-HIV controls. Calnexin shows cell contamination

Prior to the isolation of neuroexosomes, we attempted to isolate total exosomes from plasma. Extracellular vesicles in the plasma contain not only exosomes but also apoptotic bodies and macrovesicles. Therefore, according to the MISEV2018 (Minimal Information for Studies of Extracellular Vesicles 2018) guidelines (https://www.isev.org/misev2018) (Théry et al. 2018), we determined whether total exosomes could be separated based on exosome morphology, size, and protein markers. The guidelines recommend electron microscopy for observing exosome morphology. Total exosomes isolated from non-HIV control plasma and plasma from PLWH diagnosed with HAND (HAND PLWH) were examined through transmission electron microscopy. The results showed that only spherical vesicles and no other impurities were present in the total exosome samples (Fig. 1a, b). Next, the guidelines recommend measuring the size and number of vesicles and particles in the sample to determine whether exosomes that meet the criteria (50–150 nm in diameter) have been properly obtained. Therefore, we measured the size of the exosomes and counted the number of particles using a NanoSight LM10 system (Malvern Panalytical). The median diameter of the total exosomes from the non-HIV-infected samples was 163 nm, and 3.35 × 10^8^ particles/mL were detected (Fig. 1c). The median diameter of total exosomes from HAND PLWH was 132 nm, and 3.94 × 10^8^ particles/mL were detected. The total exosomes from HAND PLWH tended to be smaller in size and more numerous than those from non-HIV controls (Fig. 1d). The guidelines recommend that the absence of cellular components and the detection of exosome-specific protein markers can be demonstrated using western blotting. The surface antigens CD9, CD81, and CD63, which belong to the tetraspanin family, are exosome markers. In contrast, calnexin is abundant in the cytoplasm and is detected when cells are contaminated. Investigation of the optimal conditions for western blotting was performed using proteins extracted from total exosomes for CD9, CD81, and CD63 and proteins extracted from PBMCs for calnexin (Supplementary Fig. 1, Table 3). CD9, CD81, and CD63 were detected in the non-HIV-derived total exosomes (Fig. 1e). In contrast, calnexin was negative, indicating that no cells were present in the exosome samples. These results indicate that total exosomes of high purity were extracted.

### L1CAM-positive neuroexosomes were isolated from total exosomes

**Fig. 2:**
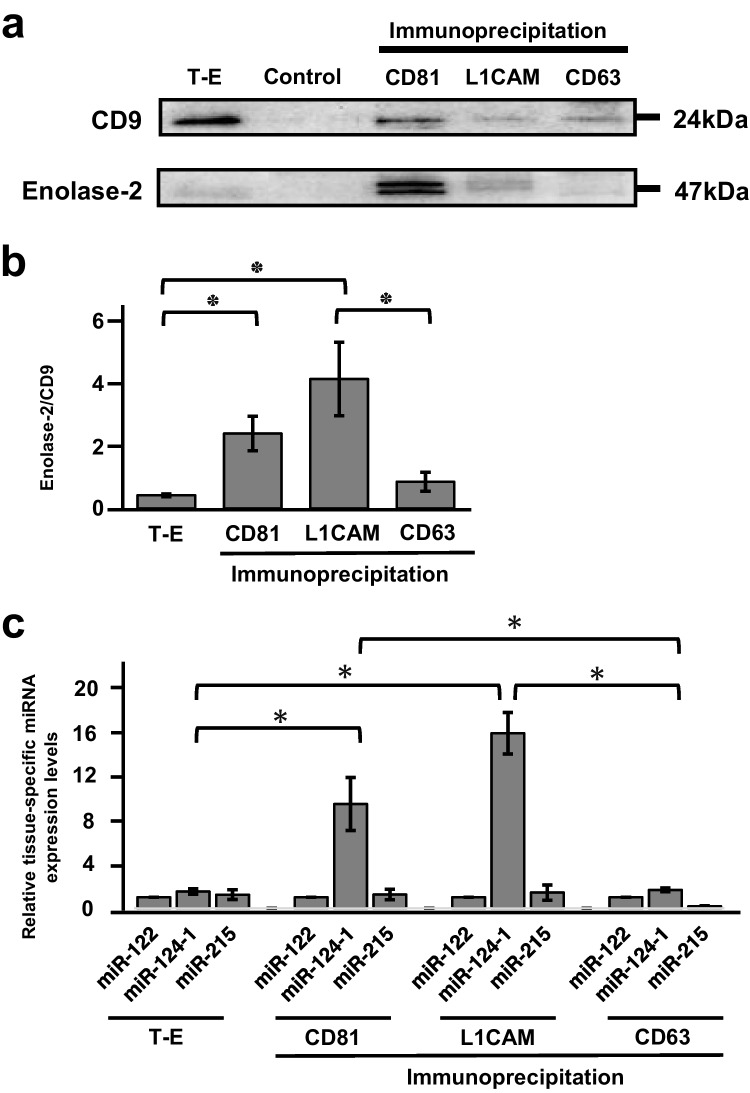
CD81- and L1CAM-positive exosomes expressed the neuronal markers enolase-2 and miR-124–1. **a** CD9 and enolase-2 detection using western blotting. CD9 is a panexosome marker. Enolase-2 is a neuroexosome marker. T-E: total exosomes isolated from non-HIV controls, Control: exosomes immunoprecipitated by control IgG, CD81: exosomes immunoprecipitated by anti-CD81 antibody, L1CAM: exosomes immunoprecipitated by anti-L1CAM antibody, CD63: exosomes immunoprecipitated by anti-CD63 antibody. **b** Enolase-2 expression levels in total exosomes isolated from non-HIV controls (*n* = 5), CD81-positive exosomes (*n* = 5), L1CAM-positive exosomes (*n* = 5), and CD63-positive exosomes (*n* = 5). The intensity of the enolase-2 signal detected by western blotting was normalized to the intensity of the CD9 signal. *p < 0.05. **c** Expression of tissue-specific miRNAs in total exosomes (*n* = 5), CD81-positive exosomes (*n* = 5), L1CAM-positive exosomes (*n* = 5), and CD63-positive exosomes (*n* = 5). miR-122: liver marker, miR-124–1: neuronal marker, miR-125: intestinal organ marker. *p < 0.05

To isolate neuroexosomes from total exosomes, immunoprecipitation was performed using antibodies against the surface antigens of neural cells in total exosome isolated from non-HIV controls. MISEV2018 identified CD9, CD81, and CD63 as exosome surface markers. The Human Protein Atlas (https://www.proteinatlas.org/) was used as a reference for protein tissue specificity. Different expression patterns were observed for CD9, CD81, and CD63 in different organs. CD9 was universally expressed in all organs, CD81 was mainly expressed in the CNS, and CD63 was expressed in the digestive system, with no expression in the CNS (Supplementary Fig. 2). Therefore, we used CD9 as a universal marker of exosomes, CD81 as a marker of neuroexosomes, and CD63 as an exosome marker for organs not in the CNS. L1CAM, which has been used to isolate neuroexosomes (Goetzl et al. 2015), is abundantly expressed in the CNS (Supplementary Fig. 2). L1CAM was also used as a neuroexosome marker in addition to CD81. IgG served as a negative control for immunoprecipitation. Enolase-2, which is expressed in neural tissue (Schmechel et al. 1978), was detected in CD81- and L1CAM-positive exosomes through western blotting (Fig. 2a). Enolase-2 was not detected in the control IgG- or CD63-positive exosomes. CD9 was detected in all exosome samples except for the control IgG. After normalizing the variation in CD9 expression between the samples, L1CAM-positive exosomes showed greater levels of enolase-2 than total exosomes (Fig. 2b, p < 0.05, Supplementary Table 2). CD81-positive exosomes also exhibited greater enolase-2 expression than total exosomes (*p* < 0.05). In contrast, enolase-2 expression in CD63-positive exosomes was not significantly different from that in total exosomes (p > 0.05).

Exosomes contain miRNAs, and the expression of miRNAs varies among organs (Lagos-Quintana et al. 2002). miR-122 is expressed in the liver, miR-124–1 in neural cells, and miR-215 in intestinal organs (Gao et al. 2011; Gotanda et al. 2016; Jopling 2012; Lagos-Quintana et al. 2002). Therefore, to examine the expression of tissue-specific miRNAs in immunoprecipitated exosomes, miRNAs were extracted from exosomes, and miRNA expression levels were subjected to relative quantification through real-time PCR. The quality of the miRNAs extracted from the exosomes using a bioanalyzer showed that no cell-derived 18S rRNAs were detected, and only miRNAs were extracted (Supplementary Fig. 3a-h). The real-time PCR results showed that the neuronal marker miR-124–1 was highly expressed in CD81- and L1CAM-positive exosomes (Fig. 2c). Similarly to those in total exosomes, the expression of the liver marker miR-122 and intestinal marker miR215 in CD81- and L1CAM-positive exosomes was low. In contrast, the expression of miR-122, miR124-1, and miR-215 in CD63-positive exosomes was as low as that in total exosomes. These results suggest that CD81- and L1CAM-positive exosomes are likely derived from neural cells.

The CD81-positive exosomes were assumed to be able to isolate neuroexosomes to the same extent as L1CAM-positive exosomes. However, because the expression of enolase-2 and miR-124–1 was greater in L1CAM-positive exosomes than in CD81-positive exosomes, L1CAM-positive exosomes were used in subsequent experiments on neuroexosomes.

### Identification of miRNAs with increased or decreased expression in HAND PLWH

**Fig. 3:**
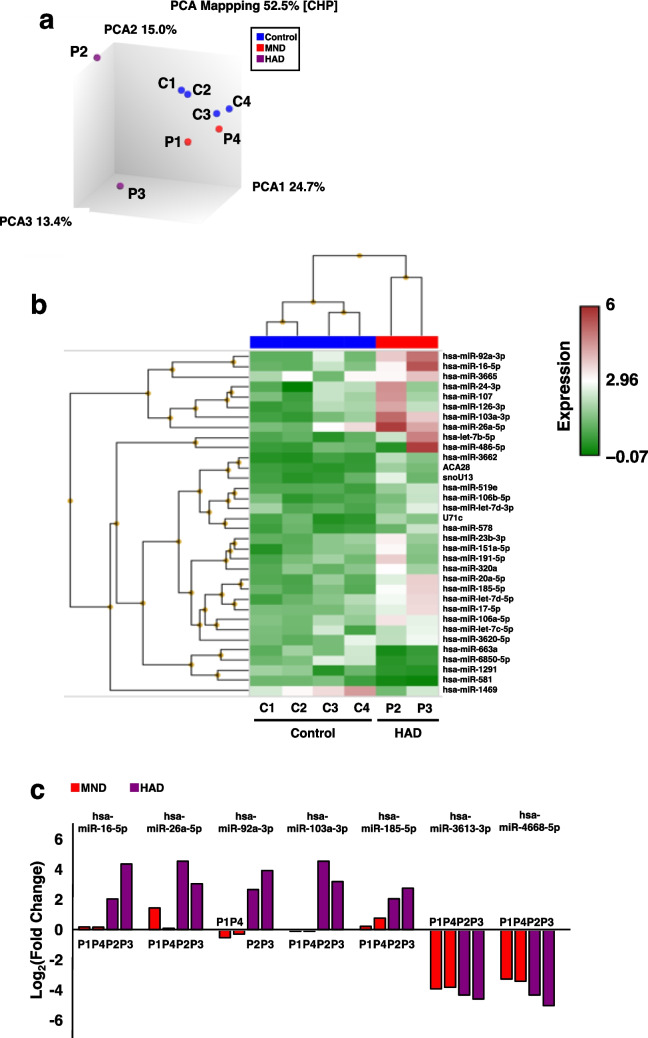
Transcriptome analysis using miRNA microarrays detected miRNAs with variable expression levels in PLWH diagnosed with HAD. **a** Principal component analysis between non-HIV controls (*n* = 4), PLWH diagnosed with MND (*n* = 2), and PLWH diagnosed with HAD (*n* = 2). **b** Heatmap of miRNAs with differential expression between the non-HIV controls and PLWH diagnosed with HAD (*p* < 0.05; fold change > 2). The shade of each color reflects the degree of increase or decrease in expression. **c** Log2 (fold change) of hsa-miR-16-5p, hsa-miR-26a-3p, hsa-miR-92a-3p, hsa-miR-103a-3p, hsa-miR-185-5p, hsa-miR-3613-3p, and hsa-miR-4668-5p with large differences in expression (fold change > 4) compared to C4 in the PLWH diagnosed with MND and HAD. MND: red, HAD: purple

To identify HAND biomarkers, we compared the expression levels of miRNAs in the neuroexosomes of HAND PLWH (*n* = 4) and non-HIV controls (*n* = 4) using a microarray and extracted miRNAs with higher or lower expression levels in HAND PLWH. To identify miRNAs whose expression varied with HAND severity, two of the four HAND PLWH were classified as MND (moderate), and two were classified as HAD (severe). The similarity in miRNA expression patterns between the samples was assessed using principal component analysis (Fig. 3a). The miRNA expression patterns were similar between the four non-HIV controls and two PLWH diagnosed with MND but differed significantly between the two PLWH diagnosed with HAD. Fifty-one miRNAs with significant differences (fold change [FC] > ± 1.6, *p* < 0.05) in miRNA expression between the non-HIV controls and PLWH diagnosed with HAD and MND were extracted and heat-mapped to visualize miRNA expression (Fig. 3b). The heatmaps were similar between the non-HIV controls and PLWH diagnosed with MND, but the HAD group showed higher miRNA expression than both of these groups. Seven miRNAs whose expression levels were significantly altered (FC > 4, FC < − 4) in the HAD group compared to those in the non-HIV control group were identified (Fig. 3c). In the HAD group, hsa-miR-16-5p, hsa-miR-26a-3p, hsa-miR-92a-3p, hsa-miR-103a-3p, and hsa-miR-185-5p were upregulated, whereas hsa-miR-3613-3p and hsa-miR-4668-5p were downregulated.

### miRNAs identified in HAND PLWH regulate the expression of genes related to microtubule mobility and endocytosis

**Fig. 4:**
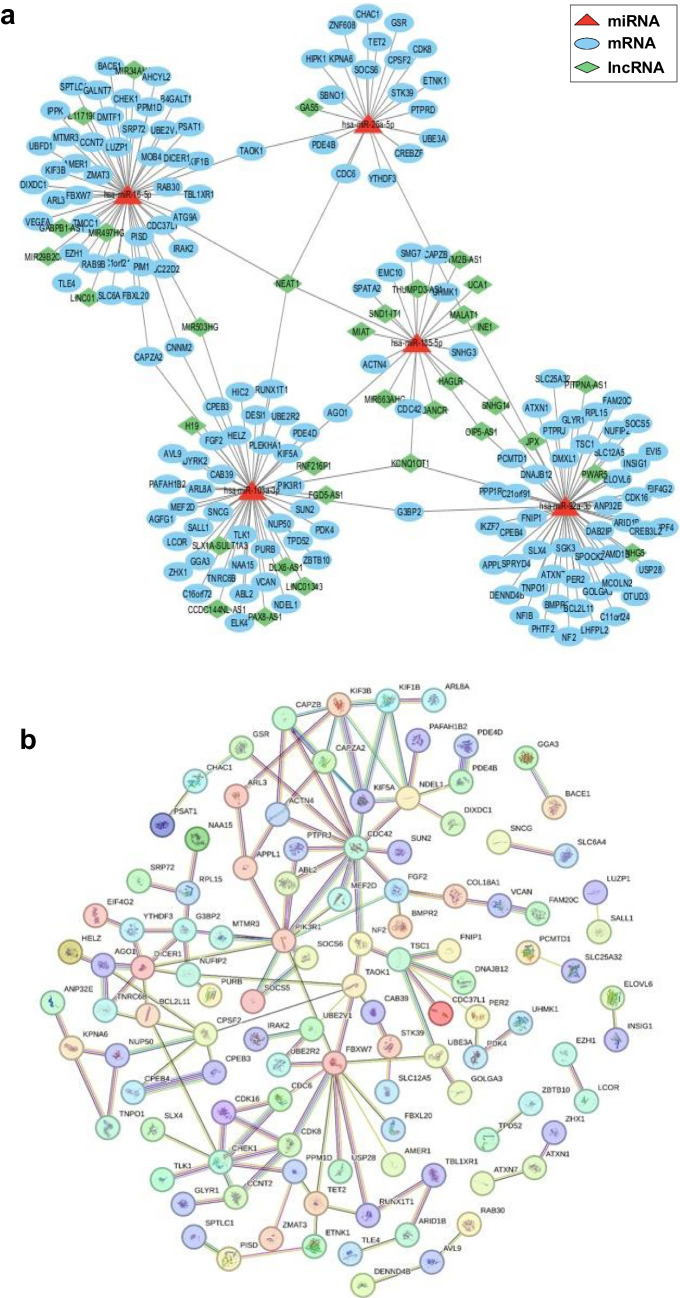
ceRNA network and PPI network. **a** ceRNA network of hsa-miR-16-5p, hsa-miR-26a-3p, hsa-miR-92a-3p, hsa-miR-103a-3p, and hsa-miR-185-5p. miRNA (red triangle), target mRNA (blue circle), and target IncRNA (green square). **b** PPI network of 158 mRNAs interacting with hsa-miR-16-5p, hsa-miR-26a-3p, hsa-miR-92a-3p, hsa-miR-103a-3p and hsa-miR-185-5p. The colored lines indicate PPIs. Light blue indicates database-supported associations, purple indicates experimentally demonstrated associations, red indicates fusion genes, yellow‒green indicates associations supported by previous studies, green indicates gene neighborhoods, blue indicates cooccurring genes, and black indicates coexpressing genes. Nodes with protein structures indicate proteins with known or predicted 3D structures. Empty nodes indicate proteins with unknown 3D structures

miRNAs interact with intracellular mRNAs and long coding RNAs (lncRNAs) to regulate translation as competing endogenous RNAs (ceRNAs). Using miRNA target prediction databases (TargetScan, miRWalk, miRDB, ENCORI, and miRTarBase), hsa-miR-16-5p, hsa-miR-26a-3p, hsa-miR-92a-3p, and hsa-miR-103a-3p were identified as mRNA targets. The RISE (Gong et al. 2018) and ENCORI databases were consulted to identify the miRNA‒target lncRNAs. The results identified 158 mRNAs and 34 lncRNAs as targets of hsa-miR-16-5p, hsa-miR-26a-3p, hsa-miR-92a-3p, hsa-miR-103a-3p, and hsa-miR-185-5p. The ceRNA networks for these 158 mRNAs, 34 lncRNAs, and 5 miRNAs were drawn in Cytoscape, revealing a network of 162 miRNA–mRNA interactions and 45 lncRNA–miRNA interactions (Fig. 4a, Table 5).

**Table 5:** Mirnas and experimentally demonstrated target mRNAs

miRNAs	target mRNA
hsa-miR-16-5p	AHCYL2 / AMER1 / ARL3 / ATG9A / B4GALT1 / BACE1 / C1orf21 / CAPZA2 / CCNT2 / CDC37L1 / CHEK1 / CNNM2 / DICER1 / DIXDC1 / DMTF1 / EZH1 / FBXL20 / FBXW7 / GALNT7 / IPPK / IRAK2 / KIF1B / KIF3B / LUZP1 / MOB4 / MTMR3 / PIM1 / PISD / PPM1D / PSAT1 / RAB30 / RAB9B / SLC6A4 / SPTLC1 / SRP72 / TAOK1 / TBL1XR1 / TLE4 / TMCC1 / TSC22D2 / UBE2V1 / UBFD1 / VEGFA / ZMAT3
hsa-miR-26a-5p	CREBZF / CDK8 / TET2 / CHAC1 / STK39 / CDC6 / ZNF608 / CPSF2 / GSR / KPNA6 / UBE3A / SBNO1 / ETNK1 / SOCS6 / PDE4B / HIPK1 / TAOK1 / PTPRD / YTHDF3
hsa-miR-92a-3p	NFIB / GRAMD1B / PCMTD1 / DMXL1 / USP28 / GLYR1 / CPEB4 / EVI5 / SLC25A32 / YIPF4 / SLX4 / DAB2IP / FNIP1 / BCL2L11 / SOCS5 / TNPO1 / MCOLN2 / CREB3L2 / INSIG1 / G3BP2 / SPRYD4 / PHTF2 / SPOCK2 / LHFPL2 / DENND4B / OTUD3 / NF2 / DNAJB12 / PPP1R37 / FAM20C / NUFIP2 / C11orf24 / PER2 / ELOVL6 / PTPRJ / GOLGA3 / CDK16 / APPL1 / SGK3 / ATXN1 / ARID1B / ATXN7 / IKZF2 / EIF4G2 / TSC1 / C21orf91 / SLC12A5 / ANP32E / BMPR2 / RPL15
hsa-miR-103a-3p	AGFG1 / TPD52 / ZHX1 / SNCG / AGO1 / CPEB3 / NDEL1 / PLEKHA1 / SUN2 / NAA15 / ZBTB10 / VCAN / RUNX1T1 / DESI1 / HELZ / PDK4 / MEF2D / CAPZA2 / ELK4 / ABL2 / C16orf72 / CAB39 / TLK1 / ARL8A / LCOR / KIF5A / PDE4D / G3BP2 / GGA3 / AVL9 / PAFAH1B2 / TNRC6B / UBE2R2 / PURB / FGF2 / PIK3R1 / SALL1 / CNNM2 / NUP50 / DYRK2 / HIC2
hsa-miR-185-5p	CDC42 / SMG7 / CAPZB / SPATA2 / EMC10 / ACTN4 / AGO1 / UHMK1

**Fig. 5:**
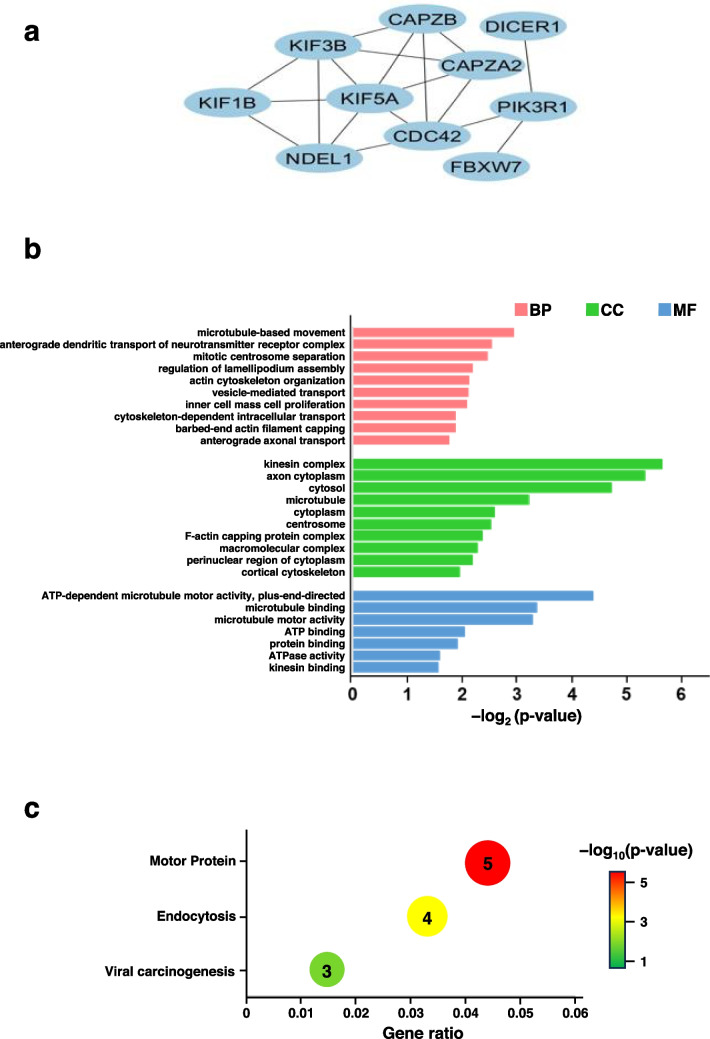
GO and KEGG enrichment analyses of 10 hub genes. **a** Predicted hub genes extracted from the PPI network. **b** GO analysis of mRNAs targeted by miRNAs in the ceRNA network. BP: biological process (pink), CC: cellular component (green), MF: molecular function (blue). **c** Bubble plot of KEGG enrichment analysis of miRNAs in the ceRNA network

Protein–protein interactions (PPIs) were analyzed using the Search Tool for the Retrieval of Interacting Genes/Proteins (STRING) database for 158 mRNAs interacting with 5 miRNAs. The PPI network comprised 110 proteins and 274 edges. Using cytoHubba Ver. 0.1, we extracted 10 hub genes from the 110 proteins identified in the PPI network (Fig. 4b, Table 6). The extracted hub gene network comprised 10 nodes and 15 edges (Fig. 5a).

**Table 6:** Experimentally demonstrated miRNA target lncRNA

miRNAs	target lncRNA
hsa-miR-16-5p	MIR497HG / MIR34AHG / GABPB1-AS1 / NEAT1 / LINC01184 / MIR29B2CHG / AL117190.1 / MIR503HG
hsa-miR-26a-5p	GAS5 / NEAT1 / MALAT1
hsa-miR-92a-3p	SNHG5 / KCNQ1OT1 / MALAT1 / SNHG14 / PWAR5 / OIP5-AS1 / PITPNA-AS1 / JPX
hsa-miR-103a-3p	LINC01343 / PAX8-AS1 / FGD5-AS1 / RNF216P1 / DLX6-AS1 / H19 / KCNQ1OT1 / NEAT1 / SLX1A-SULT1A3 / CCDC144NL-AS1 / MIR503HG
hsa-miR-185-5p	SNHG3 / HAGLR / THUMPD3-AS1 / DANCR / SND1-IT1 / NUTM2B-AS1 / KCNQ1OT1 / NEAT1 / MALAT1 / SNHG14 / OIP5-AS1 / UCA1 / MIR663AHG / MIAT/ INE1

To investigate the functions of the five miRNAs upregulated in PLWH diagnosed with HAD, Gene Ontology (GO) and Kyoto Encyclopedia of Genes and Genomes (KEGG) enrichment analyses were performed on the 10 hub genes using DAVID. GO analysis revealed that the 10 hub genes were localized intracellularly, formed kinesin and microtubule complexes, and were involved in microtubule motility, such as axonal and vesicular trafficking (Fig. 5b). KEGG analysis revealed that the 10 hub genes are associated with motor proteins and endocytosis (Fig. 5c). These results suggest that the five miRNAs identified in this study, which were upregulated in PLWH diagnosed with HAD, may regulate the translation of genes related to microtubule motility and endocytosis, which are closely associated with neuronal function.

### hsa-miR-16-5p, hsa-miR-103a-3p, and hsa-miR-185-5p are candidate HAND biomarkers

**Fig. 6:**
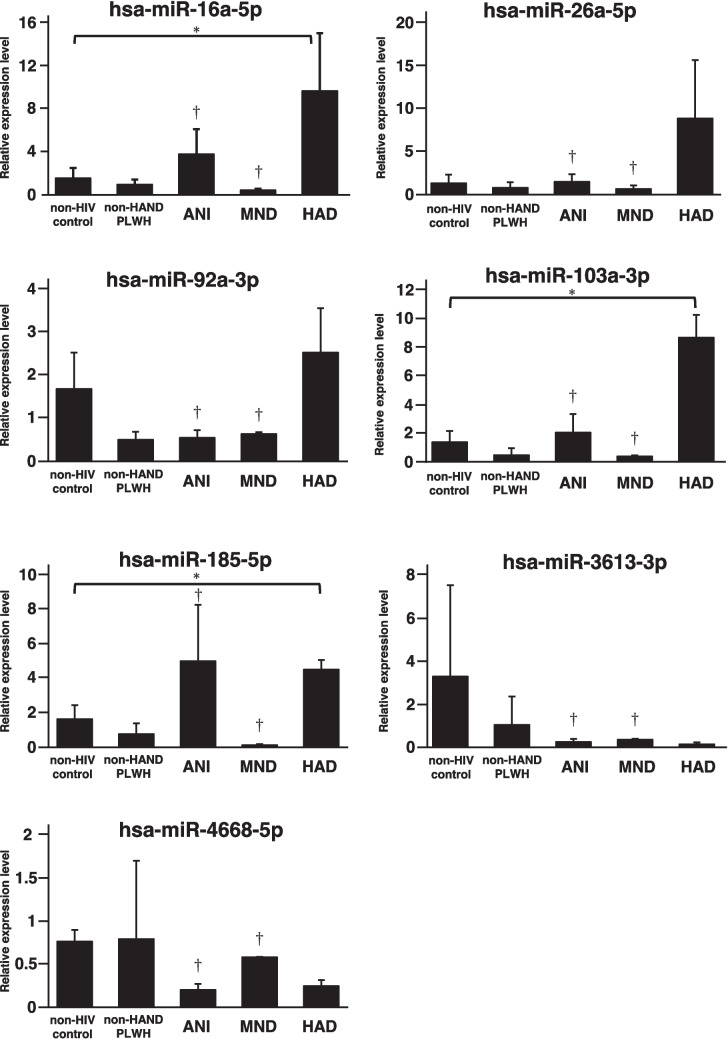
hsa-miR-185-5p, hsa-miR-103a-3p, and hsa-miR-16-5p are candidate biomarkers for HAND. Expression of hsa-miR-16-5p, hsa-miR-26a-3p, hsa-miR-92a-3p, hsa-miR-103a-3p, hsa-miR-185-5p, hsa-miR-3613-3p, and hsa-miR-4668-5p in neuroexosomes extracted from non-HIV controls (blue), non-HAND PLWH (pink), and HAND PLWH, including ANI (orange), MND (red), and HAD (purple). *p < 0.05. ^†^ shown in ANI and MND means that the results of the qPCR results of AND and MND were excluded from the statistical analysis because there were only two cases for each

The expression of miRNAs in the neuroexosomes of PLWH was examined through RT‒qPCR to determine whether the seven identified miRNAs (hsa-miR-16-5p, hsa-miR-26a-3p, hsa-miR-92a-3p, hsa-miR-103a-3p, hsa-miR-185-5p, hsa-miR-3613-3p, and hsa-miR-4668-5p) could be potential HAND biomarkers. Neuroexosomes were isolated from non-HIV controls (*n* = 5), non-HAND PLWH (*n* = 6), and HAND PLWH (*n* = 7), including ANI (*n* = 2), MND (*n* = 2), and HAD (*n* = 3). The results showed that the expression of hsa-miR-16-5p, hsa-miR-103a-3p, and hsa-miR-185-5p was significantly greater in the HAD group than in the non-HIV control group (Fig. 6, *p* < 0.05; Supplementary Table 2). Statistical analysis was not possible because there were only two cases each of ANI and MND, but the expression levels of these three miRNAs were generally the same as in non-HIV controls, except for one ANI case (P8) with hsa-miR-185-5p, which had a higher expression level. There was a trend toward increased expression of hsa-miR-26-5p and hsa-miR-92a-3p in the HAD group compared to the non-HIV control group, but this difference was not significant (*p* value > 0.05, Supplementary Table 2). The expression levels of these five miRNAs were not increased in non-HAND PLWH and were not significantly different (*p* value > 0.05, Supplementary Table 2) from those in non-HIV controls. There was a trend toward lower expression of hsa-miR-3613-3p and hsa-miR-4668-5p in the HAD group than in the non-HIV control group, but the difference was not significant (*p* value > 0.05, Supplementary Table 2). The expression levels of hsa-miR-3613-3p and hsa-miR-4668-5p in non-HAND PLWH were not decreased compared to those in non-HIV controls, and there was no significant difference between the two experimental groups (*p* value > 0.05, Supplementary Table 2). There was a trend toward decreased expression of hsa-miR-4668-5p in PLWH diagnosed with ANI, but statistical analysis could not be performed because there were only two PLWH. Neither hsa-miR-3613-3p nor hsa-miR-4668-5p expression was decreased in PLWH diagnosed with MND. Similar experiments were validated using miRNAs extracted from total exosomes. The results showed no difference in the expression levels of any of the seven miRNAs between the experimental groups (*p* value > 0.05, Supplementary Fig. 4, Supplementary Table 2).

There are known miRNAs whose expression levels in exosomes are increased in AD and PD (hsa-miR-22, hsa-miR-23a-3p, and hsa-miR-125b-5p) and in AD, PD, and VD (hsa-miR-135a) (Dong et al. 2020). The expression levels of these dementia markers were examined by RT‒qPCR using miRNAs extracted from the neuroexosomes of non-HAND PLWH and HAND PLWH. The results showed that the expression levels of these four miRNAs were not increased in HAND PLWH (Supplementary Fig. 5). These results highlight hsa-miR-16-5p, hsa-miR-103a-3p, and hsa-miR-185-5p as candidate HAND biomarkers.

## Discussion

In this study, we aimed to identify biomarkers in the peripheral blood of PLWH diagnosed with HAND. Microarray analysis revealed five miRNAs whose expression was markedly increased (hsa-miR-16-5p, hsa-miR-26a-3p, hsa-miR-92a-3p, hsa-miR-103a-3p, and hsa-miR-185-5p) and two miRNAs whose expression was decreased (hsa-miR-3613-3p and hsa-miR-4668-5p) in neuroexosomes from PLWH diagnosed with HAD. Database analysis revealed that these seven miRNAs interact with mRNAs encoding 10 hub genes. These hub genes are involved in microtubule motility and endocytosis. *CAPZA* and *CAPZB* encode proteins involved in the maturation of endocytic vesicles. Wang et al. (2021) reported that endocytosis of these genes was abnormal in double-knockout cells. *KIF1A*, *KIF3B*, and *KIF5A* are members of the kinesin superfamily that encode motor proteins specifically expressed in nerves (Niclas et al. 1994; Okada et al. 1995; Xu et al. 2018). Kinesins transport synaptic vesicle precursors, membrane vesicles, lysosomes, and other organelles in neurons (Nakata et al. 1998; Yamazaki et al. 1996) and play important roles in cell survival and morphogenesis (Kondo et al. 2012). In particular, the inhibition of *KIF1A* in cultured nerve cells results in dendrite regression (Yonekawa et al. 1998). *CDC42* encodes a protein that regulates the polymerization of cell surface actin; when *CDC42* is inhibited, endocytosis is impaired (Chadda et al. 2007). Failure of endocytosis results in the inhibition of the neurotransmitter release cycle in neurons and the uptake of extraneuronal nutrients. Therefore, if the seven miRNAs identified in this study suppress the expression of *KIF1A*, *KIF3B*, *KIF5*, *CDC42*, *CAPZA*, and *CAPZB*, these miRNAs may be involved in inhibiting the transport of cellular organelles via motor proteins and the uptake of neurotransmitters and nutrients via endocytosis, potentially leading to severely impaired neuronal function.

Previous studies have shown that hsa-miR-16a-5p, hsa-miR-26a-5p, hsa-miR-103a-3p, hsa-miR-92a-3p, and hsa-miR-185-5p target mRNAs associated with neurodegenerative diseases and psychiatric disorders (Chen et al. 2021; Peña-Bautista et al. 2022; Sabaie et al. 2022; Wang et al. 2020; Xie et al. 2022). hsa-miR-16a-5p targets *BDNF*, *NPY4R*, and *GLUD1*, which have been reported to be involved in the pathophysiology of anxiety and depression (Chen et al. 2021). hsa-miR-26a-5p and hsa-miR-103a-3p target *PTGS2* (Xie et al. 2022; Yang et al. 2018). Prostaglandins ameliorate amyloid-β-induced neurotoxicity and inhibit apoptosis in neurons (Yagami et al. 2003). *SYNJ1*, the target of hsa-miR-92a-3p, is involved in the clearance of amyloid beta, the protein responsible for AD (McIntire et al. 2012). In addition, hsa-miR-185-5p targets *SHISA7*, which interacts with GABA-A receptors localized on the membranes of GABAergic inhibitory synapses (Han et al. 2019). Among the genes encoding hsa-miR-3613-3p and hsa-miR-4668-5p, whose expression was decreased in PLWH diagnosed with HAD according to microarray analysis, hsa-miR-3613-3p is linked to epilepsy (Yan et al. 2017). Taken together, these findings suggest that the increased expression of the five miRNAs identified in neuroexosomes in this study may significantly impair neuronal function.

Of the seven miRNAs identified in this study that are significantly up- or downregulated in PLWH diagnosed with HAD, only hsa-miR-16-5p was reported to be upregulated in AD (Dong et al. 2020). Except for hsa-miR-16-5p, the other six miRNAs were not consistent with previously reported dementia miRNA biomarkers. The expression levels of miRNAs upregulated in AD/PD patients and hsa-miR-135a upregulated in AD/PD/VD patients were not increased in the PLWH diagnosed with HAND included in this study. This suggests that those PLWH were unlikely to have AD/PD/VD. On the other hand, since the expression level of hsa-miR-16-5p is also increased in AD, it is unclear whether the HAND biomarkers identified in this study can be used to distinguish HAND from dementia. To differentiate HAND from dementia, it is necessary to examine the expression levels of dementia biomarkers in addition to HAND biomarkers. To accurately differentiate HAND, the differences and similarities between HAND and dementia must be carefully elucidated, for example, by examining the expression of HAND biomarkers in the neuroexosomes of patients with dementia. Although it is highly likely that the seven miRNAs identified in this study act in combination to damage neurons, clarifying the effect of overexpressing these miRNAs in human brain neurons is necessary.

Our study has several limitations. First, of the approximately 500 PLWH treated at the hospital, only seven PLWH were diagnosed with HAND by the NP battery test, which is too small a number to properly evaluate whether the miRNAs identified in this study can be generalizable as biomarkers for HAND. To solve this problem, future experiments should be conducted with a large number of PLWH to determine the expression of the candidate HAND biomarkers identified in this study and to conduct NP battery testing on PLWH exhibiting high expression of those biomarkers. Another limitation of this study is the lack of blood samples at the time of HAND diagnosis and the inability to schedule blood draws under conditions suitable for the experiment. Although all blood samples used in this study were collected and cryopreserved for other research purposes before HAND diagnosis, the timing varied from three months to more than a year prior. Because of the variability in the timing of blood collection, it was not possible to observe a temporal increase in the expression of HAND biomarker candidates with severity, which was one of the objectives of this study. Furthermore, the backgrounds of the seven PLWH who underwent HAND were not uniform. Of the seven PLWH diagnosed with HAND in this study, two PLWH diagnosed with MND were pretreated with ART. The expression of HAND biomarker candidates did not increase in PLWH diagnosed with MND who did not receive ART. Therefore, we believe that viremia had little impact on the results of this study. A third limitation is the use of microarrays to identify miRNAs whose expression increases or decreases specifically in PLWH diagnosed with HAND. Microarrays and next-generation sequencing (NGS)-based miRNA sequencing have been used for transcriptome analysis of miRNAs contained in the exosomes of patients with dementia (Dong et al. 2020). The microarray results obtained in this study can be revalidated by comparison with previous studies conducted with microarrays (Hashemi et al. 2023) but not with those conducted with miRNA sequencing data.

In conclusion, we identified three miRNAs (hsa-miR-16a-5p, hsa-miR-103a-3p, and hsa-miR-185-5p) from neuroexosomes in PLWH diagnosed with HAND as candidate HAND biomarkers. These miRNAs target genes involved in kinesin complexes and endocytosis and are associated with neurodegenerative diseases. In addition, since the expression of these miRNAs increased before the diagnosis of HAND, it may be possible to detect neurocognitive disorders using a blood test before PLWH become aware of them.

## Supplementary Information

Below is the link to the electronic supplementary material.

Supplementary file1 (PPTX 812 KB)

Supplementary file2 (XLSX 9 KB)

Supplementary file3 (XLSX 83 KB)

## Data Availability

The data, including clinical information of HIV patients enrolled in this study, are not made public for privacy or ethical reasons. The raw miRNA microarray data analyzed in this study are registered at the DDBJ, and all researchers can access the data at the NCBI Gene Expression Omnibus (accession number: E-GEAD-846). miRNA qPCR data is provided within the supplementary information files.

## Abbreviations

HIV: Human immunodeficiency virus
PLWH: People living with HIV
ART: Antiretroviral therapy
HAND: HIV-associated neurocognitive disorder
ANI: Asymptomatic neurocognitive impairment
MND: Mild neurocognitive disorder
HAD: HIV-associated dementia
AD: Alzheimer's disease
CNS: Central nervous system
MicroRNA: MiRNA
PBMC: Peripheral blood mononuclear cell
RT‒Qpcr: Reverse transcription‒quantitative polymerase chain reaction
TEM: Transmission electron microscopy
GO analysis: Gene Ontology analysis
BP: Biological process
CC: Cellular component
MF: Molecular function
lncRNA: Long noncoding RNA
ceRNA: Competing endogenous RNA
PPI: Protein–protein interaction
KEGG: Kyoto Encyclopedia of Genes and Genomes
DAVID: Database for Annotation, Visualization, and Integrated Discovery
