# Supplementary material for: MicroRNA in neuroexosome as a potential biomarker for HIV-associated neurocognitive disorders
Source: J Neurovirol. 2025 Jan 16;31(1):56–74. doi: 10.1007/s13365-024-01241-8 (PMC11971210; doi:10.1007/s13365-024-01241-8)
Supplement: Supplementary file 1 — Supplementary file1 (PPTX 812 KB) [file 13365_2024_1241_MOESM1_ESM.pptx]

## Slide 1
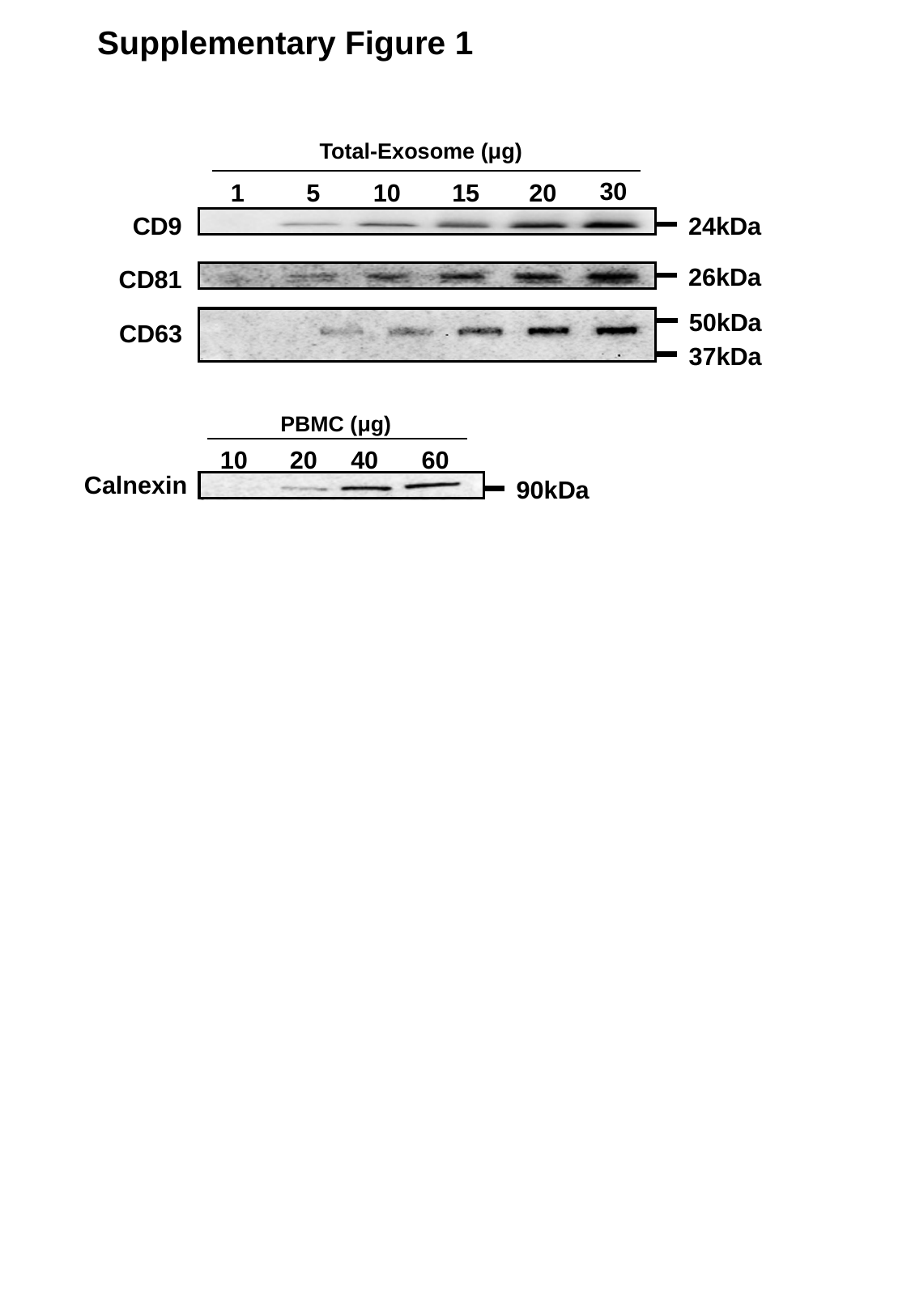

Supplementary Figure 1
Total-Exosome (μg)
30
10
15
20
1
5
CD9
24kDa
26kDa
CD81
50kDa
CD63
37kDa
PBMC (μg)
20
40
10
60
Calnexin
90kDa

## Slide 2
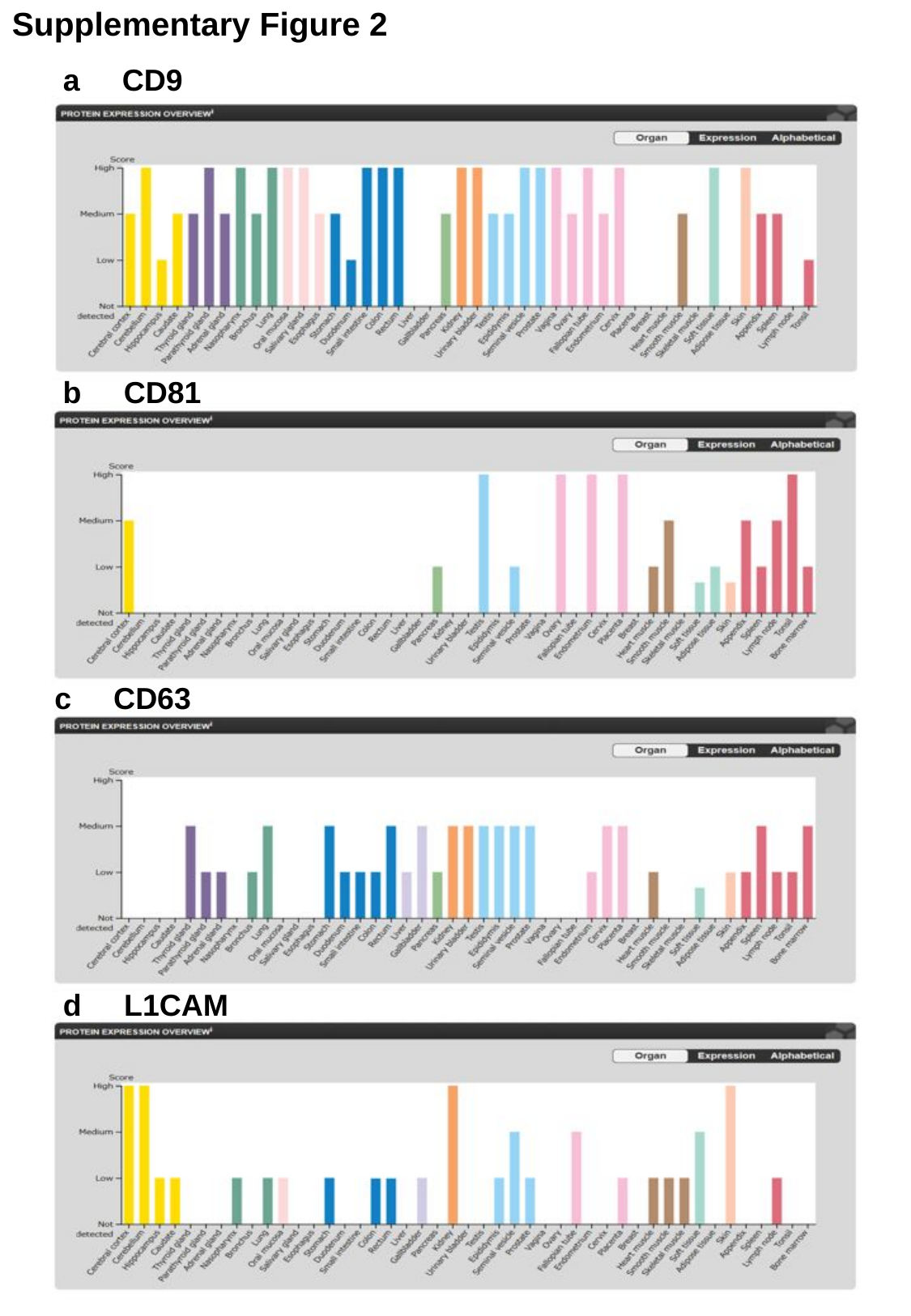

Supplementary Figure 2
a CD9
b CD81
c CD63
d L1CAM

## Slide 3
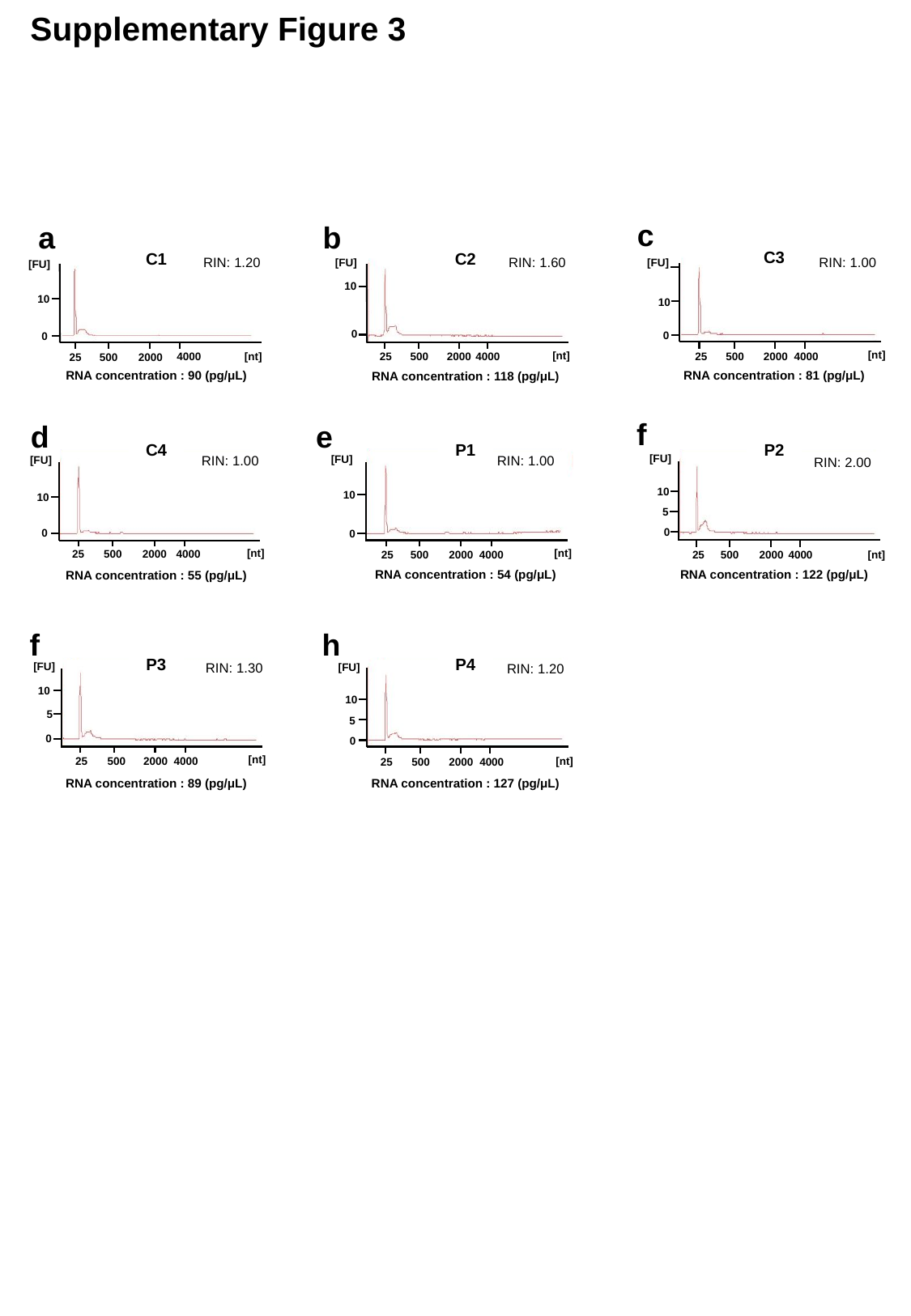

Supplementary Figure 3
c
b
a
C3
C1
C2
RIN: 1.20
RIN: 1.60
RIN: 1.00
[FU]
[FU]
[FU]
10
10
10
0
0
0
[nt]
[nt]
4000
4000
2000
4000
[nt]
2000
25
500
25
500
25
500
2000
RNA concentration : 81 (pg/μL)
RNA concentration : 90 (pg/μL)
RNA concentration : 118 (pg/μL)
f
e
d
C4
P1
P2
[FU]
[FU]
RIN: 1.00
RIN: 1.00
[FU]
RIN: 2.00
10
10
10
5
0
0
0
[nt]
[nt]
4000
25
500
2000
25
500
2000
4000
[nt]
25
500
2000
4000
RNA concentration : 54 (pg/μL)
RNA concentration : 122 (pg/μL)
RNA concentration : 55 (pg/μL)
h
f
P3
P4
[FU]
RIN: 1.30
[FU]
RIN: 1.20
10
10
5
5
0
0
[nt]
25
500
2000
4000
[nt]
4000
25
500
2000
RNA concentration : 89 (pg/μL)
RNA concentration : 127 (pg/μL)

## Slide 4
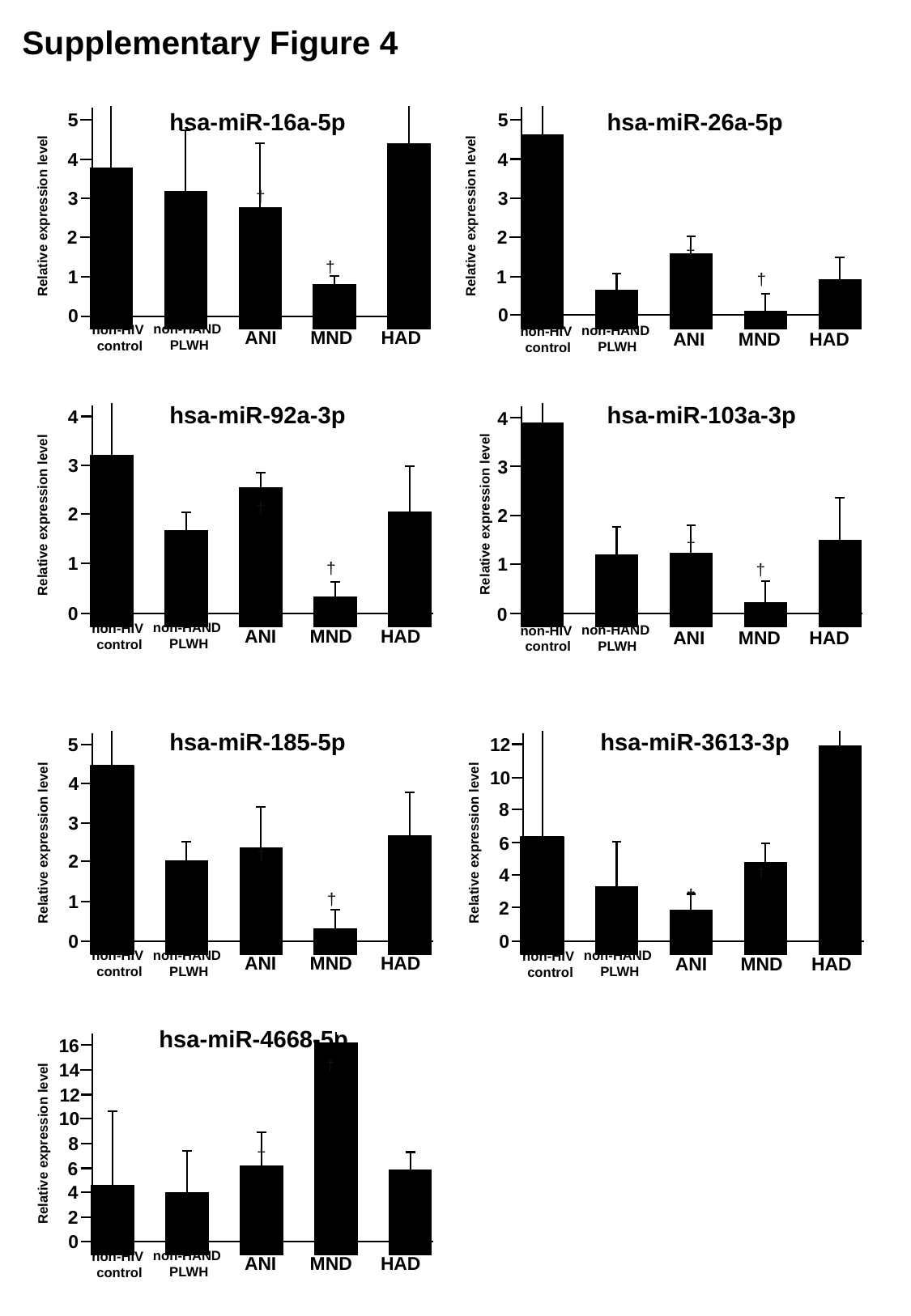

Supplementary Figure 4
### Chart
| Category | |
|---|---|
| non-HIV Control (n=5)
 | 2.168057163194586 |
| non-HAND HIV (n=6)
 | 1.8440347273669262 |
| ANI (n=2)† | 1.6330057385586372 |
| MND (n=2)† | 0.5961303632248172 |
| HAD (n=3) | 2.4875312785407853 |hsa-miR-16a-5p
### Chart
| Category | |
|---|---|
| non-HIV Control (n=5)
 | 2.1713571991946377 |
| non-HAND HIV (n=6)
 | 0.43310809711367887 |
| ANI (n=2)† | 0.8474507371912301 |
| MND (n=2)† | 0.19957957635009502 |
| HAD (n=3) | 0.5581364548253133 |hsa-miR-26a-5p
5
5
4
4
†
3
3
Relative expression level
Relative expression level
2
2
†
†
1
1
†
0
0
non-HAND
PLWH
non-HIV
control
non-HAND
PLWH
non-HIV
control
ANI
MND
HAD
ANI
MND
HAD
hsa-miR-92a-3p
hsa-miR-103a-3p
### Chart
| Category | |
|---|---|
| non-HIV Control (n=5)
 | 1.9151859557326194 |
| non-HAND HIV (n=6)
 | 1.0766681753764904 |
| ANI (n=2)† | 1.553141646994992 |
| MND (n=2)† | 0.3272606427615087 |
| HAD (n=3) | 1.279211694796562 |
### Chart
| Category | |
|---|---|
| non-HIV Control (n=5)
 | 1.8218201265336653 |
| non-HAND HIV (n=6)
 | 0.6404866498868559 |
| ANI (n=2)† | 0.6590890042408422 |
| MND (n=2)† | 0.21641521787100046 |
| HAD (n=3) | 0.7741741926311282 |4
4
3
3
†
2
2
Relative expression level
Relative expression level
†
1
1
†
†
0
0
non-HAND
PLWH
non-HIV
control
non-HAND
PLWH
non-HIV
control
ANI
MND
HAD
ANI
MND
HAD
hsa-miR-3613-3p
hsa-miR-185-5p
### Chart
| Category | |
|---|---|
| non-HIV Control (n=5)
 | 2.1090595605594755 |
| non-HAND HIV (n=6)
 | 1.0454523915617984 |
| ANI (n=2)† | 1.191000500320523 |
| MND (n=2)† | 0.2888559541726088 |
| HAD (n=3) | 1.3240889965012757 |
### Chart
| Category | |
|---|---|
| non-HIV Control (n=5)
 | 3.1515114629815466 |
| non-HAND HIV (n=6)
 | 1.8236099080210113 |
| ANI (n=2)† | 1.1856073985282054 |
| MND (n=2)† | 2.4857775303922733 |
| HAD (n=3) | 5.597851369982178 |12
5
10
4
8
3
6
Relative expression level
Relative expression level
†
2
†
4
†
†
1
2
0
0
non-HAND
PLWH
non-HAND
PLWH
non-HIV
control
non-HIV
control
ANI
MND
HAD
ANI
MND
HAD
hsa-miR-4668-5p
### Chart
| Category | |
|---|---|
| non-HIV Control (n=5)
 | 3.1139123753759077 |
| non-HAND HIV (n=6)
 | 2.7952666428979462 |
| ANI (n=2)† | 3.9856891093473985 |
| MND (n=2)† | 9.478002965306777 |
| HAD (n=3) | 3.821962361421559 |16
†
14
12
10
8
Relative expression level
†
6
4
2
0
non-HAND
PLWH
non-HIV
control
ANI
MND
HAD

## Slide 5
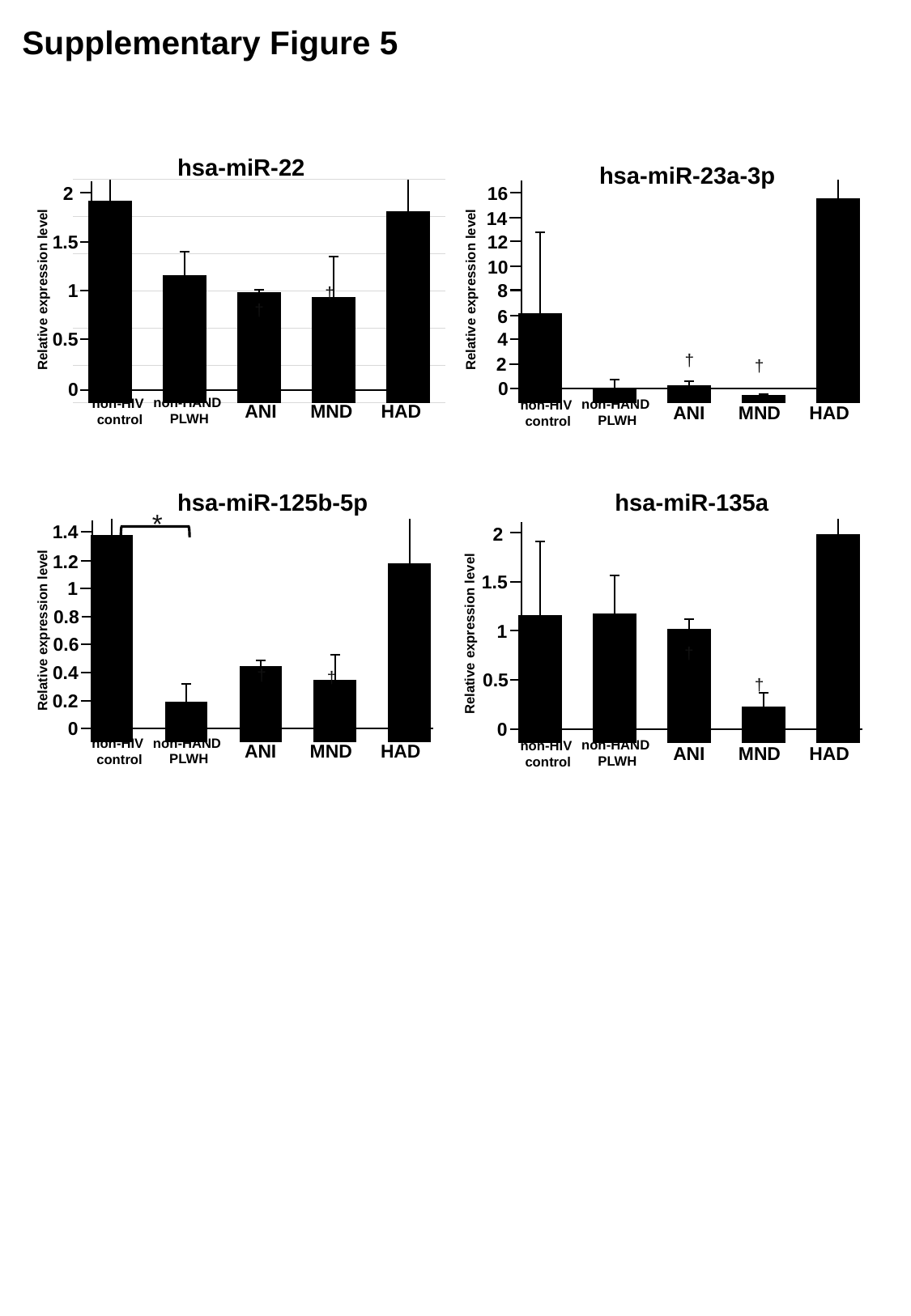

Supplementary Figure 5
hsa-miR-22
hsa-miR-23a-3p
### Chart
| Category | |
|---|---|
| non-HIV Control (n=5)
 | 1.0832656659146498 |
| non-HAND HIV (n=6)
 | 0.6828555128405118 |
| ANI (n=2)† | 0.591182997882471 |
| MND (n=2)† | 0.5653523790499047 |
| HAD (n=3) | 1.0276224643568428 |
### Chart
| Category | |
|---|---|
| non-HIV Control (n=5)
 | 3.1871442946432667 |
| non-HAND HIV (n=6)
 | 0.49491515756521265 |
| ANI (n=2)† | 0.6074588971708357 |
| MND (n=2)† | 0.26973862596397197 |
| HAD (n=3) | 7.309908526153943 |2
16
14
1.5
12
10
1
8
Relative expression level
Relative expression level
†
†
6
0.5
4
†
2
†
0
0
non-HAND
PLWH
non-HIV
control
non-HAND
PLWH
non-HIV
control
ANI
MND
HAD
ANI
MND
HAD
hsa-miR-125b-5p
hsa-miR-135a
*
### Chart
| Category | |
|---|---|
| non-HIV Control (n=5)
 | 0.6499782198924172 |
| non-HAND HIV (n=6)
 | 0.12704943819408215 |
| ANI (n=2)† | 0.23817393971400122 |
| MND (n=2)† | 0.1955562529361171 |
| HAD (n=3) | 0.5601006253928008 |1.4
### Chart
| Category | |
|---|---|
| non-HIV Control (n=5)
 | 0.5663484721628878 |
| non-HAND HIV (n=6)
 | 0.5761821004878681 |
| ANI (n=2)† | 0.5053024636770361 |
| MND (n=2)† | 0.1580786621074571 |
| HAD (n=3) | 0.9321976603130638 |2
1.2
1.5
1
0.8
1
Relative expression level
Relative expression level
0.6
†
0.4
†
†
0.5
†
0.2
0
0
non-HAND
PLWH
non-HIV
control
non-HAND
PLWH
non-HIV
control
ANI
MND
HAD
ANI
MND
HAD
